# Testing cognitive models of decision-making: selected studies with starlings

**DOI:** 10.1007/s10071-022-01723-4

**Published:** 2022-12-08

**Authors:** Alex Kacelnik, Marco Vasconcelos, Tiago Monteiro

**Affiliations:** 1 Department of Biology, University of Oxford, Oxford, UK; 2 Present Address: Department of Education and Psychology, University of Aveiro, Aveiro, Portugal; 3 Present Address: William James Center for Research, University of Aveiro, Aveiro, Portugal; 4 Domestication Lab, Konrad Lorenz Institute of Ethology, Department of Interdisciplinary Life Sciences, University of Veterinary Medicine Vienna, Vienna, Austria

**Keywords:** Choice, Decision-making, Foraging, Latencies, Response times, State-dependent valuation, Sequential choice model, Sturnus vulgaris

## Abstract

The behavioural sciences are home to controversies that have survived for centuries, notably about the relation between observable behaviour and theoretical constructs addressing out-of-sight processes in the agents’ brains. There is no shared definition for cognition, but the very existence of a thriving journal called Animal Cognition proves that such controversies are still live and help to (a) promote research on the complexity of processes leading to action, and (b) nudge scholars to restrict their cognitive models to those that can be falsified experimentally. Here, we illustrate some of these issues in a limited arena, focusing on the construction and expression of subjective value and choice. Using mainly work from our own laboratory, we show that valuation of alternatives is sensitive to options’ properties, to subject’s state, and to background alternatives. These factors exert their influence at the time the subject learns about individual options, rather than at choice time. We also show that valuation can be experimentally dissociated from the cognitive representation of options’ metrics and argue that experimental animals process options independently at the time of choice, without elaborated comparisons along different dimensions. The findings we report are not consistent with the hypothesis that preference is constructed at the time of choice, a prevalent view in human decision-making research. We argue that animal cognition, viewed as a research program at the crossroads of different behavioural sciences rather than as a debate about properties of mental life, is inspiring and solid, and a progressive and progressing paradigm.

## Background

The 25th anniversary of the journal Animal Cognition is a fitting opportunity to reflect on when and how the scientific study of cognition is both justified and pragmatically helpful in understanding animal behaviour. Here, we share some reflections, findings and theoretical ideas related to some of the work on decision-making conducted in our laboratory in the period since the birth of the journal.

Much, perhaps most, adaptive behaviour, includes sensitivity to information that is only relevant within individual lifespans. Such information is acquired and processed deploying mechanisms evolved under natural selection across generations. For this reason, articulating research on learning and decision processes with the logic of evolutionary adaptation is at the core of animal cognition research. Through their lives, animals accumulate experience in which their own behaviour is associated with specific outcomes, and make decisions by choosing between feasible actions under the influence of such information. This much is uncontroversial and fits well diverse aspects of laboratory-based associative learning research, but normative understanding and modelling of how information is acquired and deployed follows different rationales across disciplines. Ecology, evolutionary biology, neuroscience, economics and experimental psychology, all have their own theoretical and empirical frameworks to design and test models of information acquisition and decision-making in living organisms, and the science of animal cognition has much to gain by placing itself at the crossroads of these approaches.

As a general framework, rather than modelling the generation of action as directly mapped to physical properties of potential targets, as is the norm in applications of optimality in behavioural ecology, we assume that stimuli identifying reward sources acquire subjective value through learning, and these (“remembered”) values determine action. This has a parsimony cost, because we include in our models cognitive entities such as cognitive representations and putative choice algorithms, that are not directly observable, but it provides a suitable framework to distinguish the learning circumstances from those of the expression of preferences.

The distinction between objective contingencies and their subjective impact can already be found in Daniel Bernoulli’s writing about human preferences (Bernoulli 1954, page 24; see also Stearns 2000). In 1738, he wrote that “The determination of the value of an item must not be based on its price, but rather on the utility it yields. […] utility […] is dependent on the particular circumstances of the person making the estimate […] A gain of a thousand ducats is more significant to a pauper than to a rich man, though both gain the same amount.*”* He was arguing that when trying to understand preferences, one cannot just use the absolute physical parameters of the available options, but must consider what those properties mean for the person (their utility). This introduces at once many topics that are still with us today in both human and other species research, including issues to which the field of animal cognition can contribute.

## On the concept of utility

Our first topic is the concept of utility itself, and how it is handled across fields. In microeconomics theory, utility is defined as the function that is maximised by an agent’s preferences. This definition does not attribute a priori significance to substantive variables such as money, food, or reproductive success, nor is concerned with how an agent may perceive or represent its environment. In fact, “for the purpose of constructing a theory of consumer [rational] choice, not only the measurement of utility, but the very concept itself, is unnecessary. As we have seen, we can base a theory on the concepts of choice and indifference, and *nothing more is needed* for the theory than the set of indifference curves (or surfaces) with their assumed properties” (Gravelle and Rees 2004, p. 16. Emphasis in the original). This is enough to support a self-consistent theory of rational behaviour, but does not promote biological understanding of the shape of indifference curves themselves, nor concerns the cognitive operations that generate behaviour.

The closest equivalent of utility in behavioural ecology is fitness, itself a non-trivial theoretical construct (for complexities of the fitness concept see Grafen 2009, 2014). For instance, in Optimal Foraging Theory, researchers make hypotheses about the environment, about the repertoire of available actions, and about the fitness consequences of each of these actions in that environment. Behaviour is then predicted by ranking the actions in the repertoire according to their fitness consequences in that environment (Kacelnik and Cuthill 1987; Stephens and Krebs 1986; Vasconcelos et al. 2017). When data do not fit the predictions, one or more of the hypotheses is revised. Notice that, in contrast with microeconomic models, the equivalent of utility (the maximand of behaviour) is not inferred from revealed preferences, but predicted through their hypothetical trans-generational (evolutionary) consequences.

The relevance for the present discussion is that, in common with microeconomics’ utility, but not with everyday intuition, this version of utility (i.e., of what behaviour maximises, including preference in choices) does not imply a cognitively represented goal; in this research program cognition is a late-coming guest. Information-processing algorithms capable of generating the predicted behaviour are sometimes modelled, but this is done neither by assuming that the subject has fitness as a goal nor by describing its preferences, but by working out rules (strategies) that would produce optimal consequences. For instance, Houston et al. (1982) and McNamara and Houston (1985) modelled learning processes capable of behaving quasi-optimally in idealised foraging situations, but not by extrapolating from experimental results or by implementing results of previous research on animal learning, but by testing in silico which rules would converge to optimal behaviour. It is frequently argued that behavioural ecologists are concerned with functional, rather than proximate accounts of behaviour, but testing the predictive power of functional models is very hard, primarily because of the stochastic complexity of natural environments and of the heredity and development of behaviour. In practice, deviations between theoretical predictions and empirical descriptions are often accommodated by post hoc arguments about assumed cognitive processes, i.e. proximate mechanisms.

It may have become clear so far that in the course of the two and a half decades since the foundation of Animal Cognition we have become inclined to give more weight to research aimed at unravelling the cognitive processes behind observed behaviour. This has led us to develop and test models in which cognitive representations and their interactions play a decisive role. Under this view, leaving aside cognitive mechanisms when modelling behaviour emulates conchology, i.e., the branch of malacology that studies molluscs after discarding their soft parts.

A further important point about Bernoulli’s quote is his neglect of state-dependence. If identical physical rewards are not worth the same to a pauper as to a rich man, and paupers and rich men can swap places as a consequence of unstable life contingencies, then to understand choice we need to know the state of each agent. Further, if the consequences of choices are both state-dependent and learned by experience, we may ask whether the state that predicts behaviour is that at the time of learning or at the time the preference is expressed. If learning shapes preferences, then the former is to be expected. This is at odds with the influential view that preferences are constructed (and only exist) at the time of choice, when the agent judges relative, rather than absolute, properties of each alternative (Lichtenstein and Slovic 2006; see also Warren et al. 2011). Of course, by definition “preference” involves more than one alternative, but assignment of value can occur earlier, when options are experienced on their own, rather than in choices. Let us expand on this concept.

One framework can be that agents remember the physical parameters of each option, and, when more than one option is present, compare them along their dimensions to construct a ranking and make a choice. For instance, an agent may remember that two actions result each on a typical amount of and kind of food, and if forced to choose, compares the predicted consequences of the two actions in terms of two dimensions, amount and palatability, to rank them. This framework is often invoked to argue that some violations of economic rationality occur because different dimensions are given different weights when constructing preference at the time of choice (for examples, see Bateson et al. 2003; Nachev et al. 2021; Sánchez-Amaro et al. 2019).

Alternatively, the agent can assign value to each option by combining its attributes whenever it experiences it, even if there is no choice involved. Then, if a choice presents itself, the options’ values can be ranked through fast and simple processes, as we discuss below. Under this hypothesis, the combination of attributes such as amount or palatability, occurs at learning time, more likely on sequential encounters wherein only one alternative is available. Preference, even if not expressed because it is a choice-dependent concept, is latent (has been constructed) already before the agent has experienced any choice. In this case, to predicate that preference is constructed at choice time would be unhelpful. It has been said that “kicks in behinds” must have existed in the mind of God before He created kicks and behinds; more modestly, we argue that preferences can exist in latent form in deciding agents before they ever choose, so that when this happens, preferences are not constructed, but just expressed. As we shall illustrate, our studies, chiefly performed using starlings, lead us to believe that the latter is a more accurate account of how experimental animals act towards and choose between alternative opportunities, at least in laboratory contexts.

In summary, our stand is that hypotheses about cognitive processes, although not directly observable, are an essential component of behavioural research, and that a confluence between allied sciences dedicated to the understanding of decision processes is at the same time necessary, fun, and rich in consequences. A research program sensitive to these reflections would include assumptions of rationality or utility maximisation derived from economics, mechanistic discoveries of cognitive and behavioural experimental psychology, and formal analyses of the relation between experience and fitness.

In the next sections we revisit the problem of option valuation in the context of choice, briefly discussing (a) how and when state affects valuation; (b) how context affects valuation; (c) how valuation can be measured; and (d) how values interact at the time of choice.

## State-dependent valuation learning

**Fig. 1:**
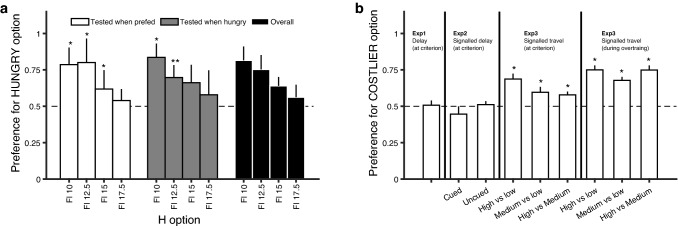
State-dependent valuation learning. **a** Preference and state. Starlings previously trained to peck for food at one stimulus (H) while hungry and a different one (PF) when pre-fed were given choices between the stimuli, while being in either state. PF’s delay to food was always 10 s, but H’s was 10, 12.5, 15 or 17.5 s, according to treatment. Data plotted separately according to testing state (white and grey bars) or pooled (black bars). The birds preferred stimuli previously experienced while hungrier, but were sensitive to how much loss (extra delay) this preference caused. Adapted with permission from Pompilio and Kacelnik, Animal Behaviour (2005)—Fig. 2—https://doi.org/10.1016/j.anbehav.2004.12.009. **b** Preference and cost. Starlings were trained separately with options that differed in the event preceding the outcome-triggering response. Differences were in either the delay (Exps. 1 and 2) or the amount of work (Exp. 3—at criterion and after extensive training). After training, they were given choices between signals for the outcome-triggering responses without having to wait or work for it. In Experiments 1 and 2, there were two options differing in delay, while in Experiment 3, there were three options differing in a required number of travel flights, leading to three different pairwise choices. The birds were indifferent when treated with delays, but preferred outcomes that were typically costlier when treated with effort. Bars represent the between-subjects average (± SEM) of individual proportions of choices. Asterisks indicate a significant (*P* < 0.05) difference from random. Adapted with permission from Aw, Vasconcelos and Kacelnik, Animal Behaviour (2011)—Fig. 4—https://doi.org/10.1016/j.anbehav.2011.02.015

We have presented the hypothesis that preferences in choice depend on the state-dependent utility experienced when an animal becomes acquainted with a potential food supply, regardless of whether this happens through choices or in sequential encounters. We have tested this idea in a diversity of experiments that involved, so far, starlings (Aw et al. 2011; Kacelnik and Marsh 2002; Pompilio and Kacelnik 2005), pigeons (Vasconcelos and Urcuioli 2008), fish (Aw et al. 2009), and grasshoppers (Pompilio et al. 2006), with consistent results across these distant taxa. The protocols were of course adjusted to each species, but the general idea was the same: to first cause sequential (one at a time) encounters with potential food sources, manipulating the subject’s own state so that one option was met when the experienced benefit of the outcome was greater than in encounters with the alternative. State was manipulated either by changing the amount of work required to gain a reward (Aw et al. 2011; Kacelnik and Marsh 2002) or by varying the state of deprivation (Aw et al. 2009; Pompilio and Kacelnik 2005). Once training had occurred, the animals faced choices in either of the states of deprivation (Fig. 1a), or between sources typically associated with different effort (Fig. 1b).

At the time of measuring preference, in most cases, the subjects had not been rewarded for specific choice behaviour. Preference for any option was higher when subjects had been in a leaner state or paid greater work for that option during learning, consistently with Bernoulli’s original statement. In contrast, state at the time of choosing had no effect on the level of preference. We refer to these findings as State-Dependent Valuation Learning, or SDVL, and—in spite of its simplicity—believe that the result is likely to be very widespread and significant, as it argues strongly against the notion that preference is constructed at the time of choice. SDVL leads to paradoxical preferences in many experimental situations, but is likely to be adaptive in natural circumstances, when fitness benefits correlate with hunger or scarcity (hence effort). The enhanced value of costlier items is equivalent to the paradoxical “sunk cost” observations in humans (see for instance Kacelnik and Marsh 2002; Navarro and Fantino 2005). The preference for costlier items in animals has also been named “work ethics” (Clement et al. 2000), but it should be noticed that in these experiments animals do not choose to work harder, but rather to get for free the typical consequences of having invested greater effort, which seems the opposite of expressing a preference for hard work under the belief that effort carries an ethical merit or has a moral value.

## The context dependence of value

**Fig. 2:**
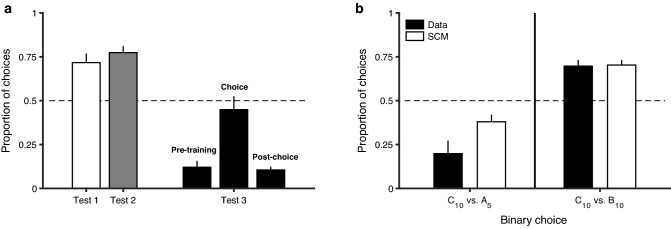
Context-dependent valuation. **a** Starlings that had previously experienced sequential encounters with options A_(5 s)_ and B_(10 s)_ in one context and with C_(10 s)_ and D_(20 s)_ in another were given choices of C_(10 s)_ Vs. B_(10 s)_ in Test 1, and of A_(5 s)_ vs. C_(10 s)_ in Test 2, with suffixes indicating the delay to food after choosing a given stimulus. In Test 3, starlings were trained with A_(5 s)_ and B_(10 s)_ in one context and with C_(14 s)_ and D_(28 s)_ in another, and were then given choices of B_(10 s)_Vs. C_(14 s)_. Test 1 shows that when delay was equated, they preferred the option with a history of better rank. Test 2 shows that when ranks were equated, they preferred the shorter delay option. Test 3 shows that when delay and rank were counterposed the effects competed, and the birds were indifferent. The Pre-training and Post-choice bars around Test 3 show that delays were discriminated: when B_(10 s)_ and C_(14 s)_ were experienced in the same context birds preferred the former, with shorter delay. Adapted with permission from Pompilio and Kacelnik, *PNAS* (2010)—Fig. 2—https://doi.org/10.1073/pnas.0907250107. **b** The black bars present a replicate of the experiment shown in panel a., and the white bars show the performance of a theoretical model (Sequential Choice Model, SCM) that addresses what happens at choice time, by predicting choices on the bases of latency to respond in encounters with single options during learning. Error bars represent one SEM. Adapted with permission from Vasconcelos, Monteiro and Kacelnik, *PLoS One* (2013)—Fig. 4—https://doi.org/10.1371/journal.pone.0064934.g004

In the previous section, we showed that the state of decision-takers at the time when they learn can be dissociated from their state at the time of expressing preferences, and that available evidence indicates that the former has greater impact. In this section, we focus on the learning environment, rather than the state of the agent. The experimental protocol in this case (Pompilio and Kacelnik 2010; see also Vasconcelos et al. 2013) was inspired by previous work in pigeons, especially by Belke (1992). As before, the learning context was experimentally dissociated from that in which preference was measured. There were four options, which in the basic protocol were identified as A_(5 s)_, B_(10 s)_, C_(10 s)_ and D_(20 s)_. The capital letters indicate an arbitrary stimulus, such as the colour of a pecking key, and the suffix is the delay for a food reward to be delivered, lapsing from the time of responding at the stimulus. During training, starlings spent time in either of two contexts, [A_(5 s)_–B_(10 s)_] or [C_(10 s)_–D_(20 s)_], with “context” being defined by the options that could be encountered. However, options at this stage were encountered sequentially, not in pairs, so that subjects did not choose between them. In the subsequent critical preference tests, animals did face pairwise choices. In Test 1, they chose between options with equal delays but different ranking (B_(10 s)_ Vs. C_(10 s)_), in Test 2, the choice was between options with equal ranking but different delay to food (A_(5 s)_ Vs. C_(10 s)_), and in Test 3 delay and ranking were counterposed (B_(10 s)_ Vs. C_(14 s)_), as explained in Fig. 2. When delays were equated (Test 1) they preferred the better ranking option, when ranking was equated (Test 2), starlings preferred the shorter delay, and when both dimensions were counterposed, they were indifferent. Sensitivity to both relative and absolute parameters can be explained by the reinforcement impact at the time of learning. In Test 1, due to the context in which B_(10 s)_ and C_(10 s)_ had typically occurred, the stimulus identifying B_(10 s)_ signalled “bad news” relative to its background, while the opposite was true for the stimulus identifying C_(10 s)_. Thus, even though both stimuli led to the same physical consequences, the hedonic impact of these consequences and hence the attached valuation of the stimuli is likely to have been greater for C_(10 s)_. In this test, the value of the stimuli at the time of choice was the same, hence could not have caused the observed preference.

The impact of ranking at the time of learning does not imply, however, that their absolute parameters are not influential: Test 2, in which two options leading to delays of 5 s and 10 s were presented, both having previously been half the delays in their alternatives, shows that when ranking of the options at learning time is not different, their absolute values determine preference at choice time. Further, Test 3 pitched relative Vs absolute values, by offering a choice between an option that was objectively better but had been the worse of a pair at learning time and an alternative that was 40% longer but had been the better option in its learning context. In this test, ranking and absolute values neutralised each other and starlings did not show any preference. These results are consistent with the view that circumstances at the time of learning are influential in the construction of preference, and that circumstances at choice time could not be responsible for the observations (see Fig. 2).

## Under no illusions: memory for temporal parameters is independent of context

**Fig. 3:**
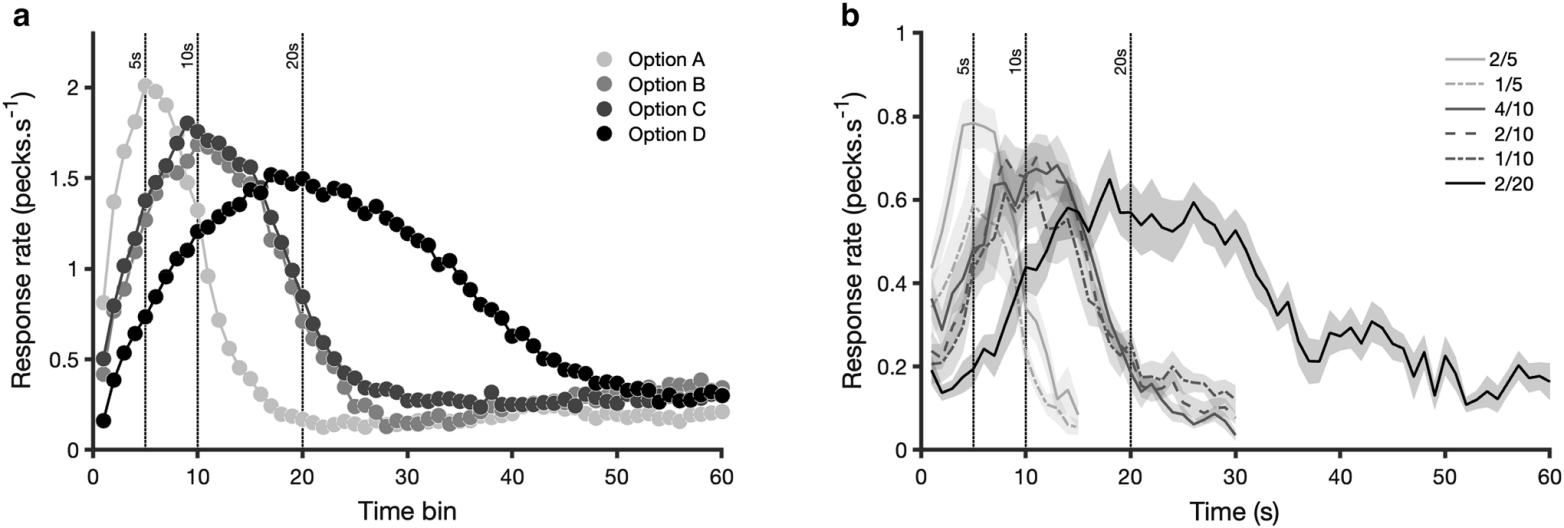
Memory for options’ metrics is independent from context. **a** Interval timing in the experiment shown in Fig. 2a. Pecking rates peaked at the veridical time of reinforcement in all four options. Notably, although starlings preferred C_(10 s)_ to B_(10 s)_ (see Fig. 2a), the temporal location of reward was unaffected. Adapted with permission from Pompilio and Kacelnik, *PNAS* (2010)—Fig. 3—https://doi.org/10.1073/pnas.0907250107. **b** In this case, starlings were trained with a mixture of 6 options encountered sequentially. Options were defined by their profitability (n pellets / t seconds of delay), as indicated in the ratios shown in the legend. Rate of responding in probe trials (mean ± SEM, *n* = 9 birds) differed between options with different delays, but not between options with different food amounts. Adapted with permission from Monteiro et al. (2020)—Fig. 1c—https://doi.org/10.1371/journal.pbio.3000841.g001

From the point of view of cognitive processing, preference for an option over another which has equal absolute properties does not necessarily imply that subjects assign option-specific hedonic value, or utility, at the time of learning. It is theoretically possible that there is no valuation, but the subjective representation of the critical metrics of each option are influenced by its learning context. In the experimental examples used to illustrate state and context effects subjects showed preference between options that were equal in absolute value. However, while options’ absolute values were equal, their subjective representations could have differed. For instance, two options with delays of 10 s could have been remembered as having shorter or longer delays, according to the subjects’ state or context. The contextual effects on preference have been successfully replicated in human subjects, and an interesting parallel has been made by Palminteri and Lebreton (2021) with the so-called “Ebbinghaus illusion”, whereby the apparent relative size of visual stimuli is influenced by context. This possibility can be rejected for our starlings’ experiments, because the behaviour of the animals allows us to measure their temporal expectations, which have been shown not to be biased (Fig. 3).

The accuracy of the animal’s knowledge of options’ metrics was measured using the peak procedure (Balcı and Freestone 2020; Catania 1970; Monteiro and Machado 2009; Roberts 1981). Figure 3a makes the case compellingly by displaying the pecking rate of starlings while waiting for the outcome of trials in encounters with the four different options described in Fig. 2a. The critical observation is that pecking rate peaks at the time the rewards would normally be delivered. In particular, pecking in options B_(10 s)_ and C_(10 s)_ peaks at 10 s. Crudely, this can be described by saying that the bird “knows” when food is due in both cases, but still prefers the option that had signalled “good news” in its learning context (see Fig. 2a). This dissociation between interval representation and valuation supports the notion of an indirectly inferred hedonic component. Figure 3b shows similar findings from Monteiro et al. (2020) wherein, for alternatives associated with the same delay but different amounts of food, response rate peaked at the same time, even though in choices starlings preferred the more profitable alternatives (i.e., the ones leading to larger amounts).

## The mechanism of choice: what happens at choice time?

The results summarised so far emphasise the intricate relation between behaviour, learning, cognition and normative models of decision-making. We have shown that, in addition to the mnemonic representation of metrics of food sources, animals store information about the hedonic impact experienced at learning time, when options become identifiable. We call this inferred impact “valuation”. This information, we claim, is highly influential when two options are met simultaneously and the animal must behave towards just one of them, namely when it expresses a preference. We argued against the view that preference is constructed at the time of choice by comparison of the remembered parameters of each option, for two main reasons. First, preference at choice time can be predicted by measures of behaviour taken when choices have not yet occurred (see below), and second, strong preference can exist between options whose metrics subjects accurately represent as equal, or even when the more delayed of two equally sized rewards is preferred due to its history (as shown in Figs. 2 and 3).

In this section, we shift our focus to the choosing stage, and explore what happens when an already informed subject (in the sense that it has already learned the properties and assigned value to each option in its environment) encounters two options simultaneously. In brief, we propose that it is possible to detect a measure of the value an animal assigns to an option independently of its preference in choices. This behavioural measure plays a similar role to that of the Willingness-To-Pay protocols in behavioural economics (Slovic 1995), because it offers a window into an agent’s valuation of options in the absence of a choice between alternatives. For the starlings, we use temporal hesitation to take options encountered sequentially, rather than in pairs or multiple sets. This temporal hesitation, latency, or response time, has two properties of significance for the present argument: (a) everything else being equal, they are shorter when the option’s objective or relative value is greater; and (b) for a given option, latencies show some distribution of durations between trials. Both properties make sense and have been corroborated repeatedly (e.g., Monteiro et al. 2020; Shapiro et al. 2008). Here, we discuss their consequences for modelling the mechanism of choice. As we shall see, these simple facts make testable predictions which are far from trivial.

The main idea here is that when two or more options are met simultaneously, the processes that generate response times in sequential encounters are deployed independently, namely without interfering with each other. No cognitive comparative evaluation takes place at choice time. This is a parsimonious starting point, and it is worth exploring how far it can take us. Under this assumption, which is the core of the Sequential Choice Model (SCM; Aw et al. 2012; Freidin et al. 2009; Kacelnik et al. 2011; Monteiro et al. 2020; Sasaki et al. 2018; Shapiro et al. 2008; Smith et al. 2018; Vasconcelos et al. 2010, 2013), if and when stimuli corresponding to options previously encountered sequentially are encountered simultaneously, each stimulus elicits a response time by sampling from its own distribution of response times in sequential encounters. The option that in that encounter yields a shorter sample receives the action, and is seen by the observer as being “chosen”. The alternative option does not generate an observable datum on that occasion. Because the distributions of response times have some spread, choice is a stochastic process in which the option whose associated latencies tend to be shorter is chosen in the majority of encounters. Of course, the less-preferred alternative occasionally wins the race, and then it is chosen and the observer records a response time. The response time observed when an option is chosen out of a pair or set of multiple options is then a biased sample from the distribution for the same option in sequential encounters, because samples at the left tail of the distribution of each option are more likely to result in a datum than unconstrained samples. The net result is that the distribution of response times observed in choices should be shorter than those observed when each option is encountered sequentially (more on this below).

There are close precedents for this rationale in the psychological literature, and it has been argued that choice is more a methodological resource of researchers than relying on special processes adapted for decision-making, and present in the animals themselves. For instance, Herrnstein (1970) argued that even when there is only one measured response (the equivalent of our sequential trials), animals still show some allocation of responding between the response being measured and the environmental background. He argued that the richer the background the lower the rate of responding to the response being measured. He further argued that when researchers orchestrate a choice by offering more than one possible response, nothing new happens, but the rates of responding compete by matching their relative values. Rate of responding is, of course, a concept that is reciprocally related to inter-response interval, and consequently to latency when one measures delay to emit a single response. Herrnstein explicitly supported the view that nothing else is necessary to understand choice behaviour when more than one option is present. In the cited paper, he writes: “It is hard to see choice as anything more than a way of interrelating one’s observations of behavior, and not a psychological process or a special kind of behavior in its own right.” This is consistent with our stand in this respect, except that we place emphasis on the fact that it is also possible to show that the subjects can remember the true properties of options even when their choice is contrary to the ranking of the parameters.

Other authors have developed race models for choice in independent but convergent ways. Logan et al. (2014) expanded models of Stop-Signal-Response-Time (SSRT), so as to relate the study of responding to single options to data on choice. SSRT models (Logan and Cowan 1984) deal with scenarios in which subjects respond to one “Go” signal and one “Stop” one. If the onset of both signals is simultaneous, and they are expressed in behaviour through a drift diffusion process, then the subject will respond or not in each trial according to which of the two processes reaches the response threshold earlier. In the extension by Logan et al. (2014) the concept is applied to choice scenarios, where each option acts as a stop signal for the competing option(s), similarly to the notion of cross-censorship between the distributions of latencies in the SCM.

**Fig. 4:**
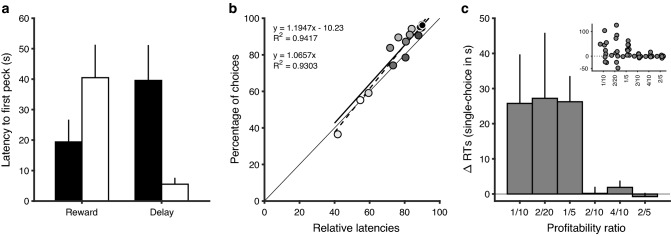
Response time as a measure of preference. **a** Starlings faced a fixed (dark) and a variable (light) option, where variability was in reward size or delay. Consistent with predicted risk aversion for amounts and risk proneness for delays, latencies to peck in no-choice trials (shown) were shorter for the fixed reward and for the variable delay option. Choice proportions in the same experiment showed risk proneness for delays but did not differ significantly from random for amounts (not shown). Adapted with permission from Reboreda and Kacelnik, *Behavioral Ecology* (1991)—Fig. 3—https://doi.org/10.1093/beheco/2.4.301. **b** Starlings were trained with 15 pairs of options differing in amount and/or delay to food, and then given choices between members of each training pair. Choice percentage (ordinate) was predicted using relative value of latencies (latency A /(latency A + latency B) × 100) to respond in probe trials (abscissa). Each treatment pair is represented by a different shading. Also included are the identity diagonal and two linear regressions. One of them (----) is unconstrained and the other (—) is constrained to pass through the origin. The *R*^2^ values are generated by the average obtained results. The fit of SCM predictions was the best among a set of candidate predictive models. Adapted with permission from Shapiro, Siller and Kacelnik, Journal of Experimental Psychology*:* Animal Behavior Processes (2008)—Fig. 8f—https://doi.org/10.1037/0097-7403.34.1.75. **c** Difference of response times in single-option trials minus response times in choice trials of the same option against all other options (*n* = 9, mean ± SEM). Inset shows individual results. For illustration, in the rare trials in which birds chose one of the two poorest option in the environment (1 pellet / 10 s) when that option was paired with any of the other 5 options, response time was ~ 25 s shorter than when the same option was met in a sequential (single-option) trial. Adapted with permission from Monteiro et al. (2020)—Fig. 3c—https://doi.org/10.1371/journal.pbio.3000841.g001

The SCM originates from the optimal foraging and risk sensitivity tradition. Reboreda and Kacelnik (1991) noticed that response times in sequential encounters were a useful metric of preference, independent of, and complementary to, the proportion of responses in choice scenarios. They were testing the hypothesis that a widespread property of perception (Weber’s Law) may cause a positive relation between the variance in outcomes of a given option and its chance of being chosen in tests in which smaller outcomes were preferable (as in delays to food), and the opposite when larger outcomes were preferable (as in amount of food). They found that choice proportions were consistent with this hypothesis, but only weakly so: experimental starlings were consistently risk prone for delays, but either risk averse or indifferent for amounts (rather than being reliably risk averse). However, when they measured response times in sequential encounters, results were reliably consistent with the hypothesis: latencies to respond in sequential encounters were significantly shorter for greater variance in delays and significantly longer for greater variance in amounts (see Fig. 4a). On this basis, they argued that preference can be measured outside choice trials, and that response time in the absence of choice may be at least as sensitive a metric of relative valuation of options as proportion of choices.

As work accumulated, we have come to believe that latency in sequential encounters is in fact a more sensitive and informative index of preference than choice proportion. The Sequential Choice Model in its present form was suggested by Shapiro et al. (2008), that studied starlings’ preferences between food sources that varied across combinations of fixed amounts and delays. They tested the fit of several different models to the data, and found that (a) latencies to respond in sequential trials were shorter the higher the profitability of an option (Profitability = Amount / Delay); (b) latency for each option was longer the higher the profitability of alternative option(s) in the environment (i.e., a context effect); and (c) for each pair of options, the best predictor of choice proportion was the relation between their latencies in no-choice trials (Fig. 4b). They called their model the Sequential Choice Model or SCM, and argued that from a normative perspective, the mechanism underlying the SCM made sense, because sequential encounters are likely to be more prevalent in nature than simultaneous ones, and latency provides a common path to integrate different factors affecting the value on each alternative. As previously alluded, the SCM makes the counterintuitive prediction that observed response times should be shorter in choices than when options are met singly, the opposite of what should be expected if elaborated cognitive work occurs at choice time. The reason why the SCM predicts that choice latencies should be shorter than sequential ones is cross-censorship between the latency distributions of the alternatives: since the model assumes that the latency distributions of each alternative in sequential encounters are sampled independently and compete for expression, shorter samples have a greater chance of winning the race and being represented in the distribution of choice latencies. This censorship effect should be more pronounced for the less-preferred option of each pair, because samples from its right tail will never win the race and will not be recorded in any choice test. The interested reader can find a computational description, analytical implementation and numerical simulations of the SCM in Monteiro et al. (2020) supporting information.

The argument that choice behaviour in simultaneous encounters should be predictable from performance in single option encounters has been supported by experimental data from several labs and subject species. Protocols include risky choice (Aw et al. 2012), context manipulations (Vasconcelos et al. 2013), sub-optimal choice preparations (Macías et al. 2021; Ojeda et al. 2018), select/reject protocols (Freidin et al. 2009), mid-session reversal protocol in pigeons (Smith et al. 2018) and environments composed of multiple options defined either by delay (Vasconcelos et al. 2010) or by profitability (i.e., amount/delay; Monteiro et al. 2020).

Testing the SCM prediction that latencies to respond should be shorter in choices presents practical difficulties. On one hand, when animals strongly value an option, its latency distribution in sequential encounters approaches minimal reaction time, and there is little or no room for a shortening effect. On the other hand, less-preferred options, whose longer mean latencies should make the difference between sequential and choice trials more detectable, are by definition seldom chosen, leading to small sample sizes. Despite these difficulties, a combination of shortening or no-change has been universally documented when comparing simultaneous with sequential decisions, and this is the opposite of the predicted temporal cost if cognitive evaluations occurred at the time of each choice. Shapiro et al. (2008), Monteiro et al. (2020)—Fig. 1c, Ojeda et al. (2018), and Mácias et al. (2021) all found reliable shortening of latencies, but Aw et al. (2012) and Vasconcelos et al. (2013) found no evidence for either shortening or lengthening. Macías et al. (2021) arranged a particularly apt procedure to test this aspect of SCM, avoiding the limited sample size problem, and found clear evidence for shortening.

It is thus likely that in typical animal experiments preference is not constructed at choice time, but is the result of valuation of options by the subjects when they learn about the parameters of each alternative. In our view, in animal experiments, there is so far no evidence for the presence of cognitive processes evolved to generate optimal outcomes in simultaneous choices. However, lack of evidence is not the same as evidence for absence, and such evidence may emerge in novel protocols. In humans, in particular, one may intuitively expect that the processes addressed here may be prevalent in situations within the realm of so-called “system 1” (Kahneman 2011), rather than in the more elaborated and slow processes assumed to be addressed by “system 2”. In experimental studies of preference with human subjects information is frequently provided by description rather than experience (for discussions of the significance of this distinction see Hertwig and Erev 2009; Kahneman and Tversky 2000; Ludvig and Spetch 2011). When options are verbally described, it is to be expected that subjects may need to reason about their properties in order to rank them, leading to a detectable lengthening of decision time respect to single option encounters. Home buyers may indeed compare separately potential homes’ merits in terms of location and quality, and may take longer when more homes or more dimensions are compared. We are not aware of studies directly addressing the dynamics of differences in latency to act in sequential versus choice encounters in humans, but such studies would clearly serve to establish closer bridges between the cognitive processes of choice of humans and other species.

## Concluding remarks

In summary, our stand is that hypotheses about cognitive processes, although not directly observable, are an essential component of behavioural research. The field of animal cognition is building a scientific program that answers the epistemological caveats that have historically been highlighted by justified critiques of mentalism. Perhaps the ideals of the cognitive revolution, who sought to replace both radical behaviourism and mentalism through a scientific approach to cognitive processes can be realised, after all, in the field of animal cognition. As evidenced in many publications in the Animal Cognition journal, whose first quarter of a century we are celebrating, this broadly defined research program is suitable to integrate contributions from a diversity of allied sciences, such as importing operational definitions of rationality derived from economics, mechanistic discoveries of cognitive psychology, rigorous empirical procedures used by behaviourists, and formal models of the relation between experience and evolutionary fitness as developed in behavioural ecology.

## Data Availability

This article containes no original data. All figure sources are included in the text.
